# “Articles of Faith” Concerning Cancer—A Platform upon Which to Unite in the Campaign of Education

**Published:** 1915-07

**Authors:** 


### "Articles of Faith" Concerning Cancer—A Platform Upon Which to Unite in the Campaign of Education.

1. That the hereditary and congenital acquirement of cancer are subjects which require much more study before any definite conclusions can be formed concerning them, and that, in the light of our present knowledge, they hold no special element of alarm.

2. That the contagiousness or infectiousness of cancer is far from proved, the evidence to support this theory being so incomplete and inconclusive that the public need have no concern regarding it.

3. That the communication of cancer from man to man is so rare, if it really occurs at all, that it may be practically disregarded.

4. That those members of the public in charge of or in contact with sufferers from cancer with external manifestations, or discharges of any kind, need at most take the same precautionary measures as would be adopted in the care of any ulcer or open septic wound.

5. That in the care of patients with cancer there is much less danger to the attendant from any possible acquirement of cancer than there is of septic infection, or blood poisoning from pus organisms.

6. That in cancer, as in all other disease, attention to diet, exercise and proper hygienic surroundings is of distinct value.

7. That, notwithstanding the possibility of underlying general factors, cancer may, for all practical purposes, be at present regarded as local in its beginning.

8. That, when accessible, it may, in its incipiency, be removed so perfectly by radical operation that the chances are overwhelmingly in favor of its non-recurrence.

9. That, when once it has advanced beyond the stage of cure, suffering in many cases may be palliated and life prolonged by surgical and other means.

10. That while other methods of treatment may, in some cases, offer hope for the cancer victim, the evidence is conclusive that surgery, for operable cases, affords the surest present means of cure.

11. That among the many advances in and additions to cancer treatment, the improvements in and extensions of surgical procedure surpass those in any other line, and fully maintain the pre-eminent position of surgical palliation and cure.

12. That there is strong reason to believe that the individual risk of cancer can be diminished by the eradication, where such exist, of certain conditions which have come to be regarded as predisposing factors in its production.

13. That some occupations, notably working in pitch, tar, paraffin, analin or soot, and with X-rays, if not safeguarded, are con ducive to the production of cancer, presumably on account of the chronic irritation or inflammation caused.

14. That prominent among these predisposing factors, for which one should be on guard, are: general lowered nutrition; chronic irritation and inflammation; repeated acute trauma; cicatricial tissue, such as lupus and other scars, and burns; benign tumors—warts, moles, nevi (birth-marks), etc.; also that changes occurring in the character of such tumors and tissues, as well as the occurrence of any abnormal discharge from any part of body, especially if blood-stained, are to be regarded as suspicious.

15. That while there is some evidence that cancer is increasing, such evidence does not justify any present alarm.

16. That suggestions which are put forward from time to time regarding eugenic, dietetic and other means of limiting cancer, should not be accepted by the public until definitely endorsed by the consensus of expert opinion. Such consensus does not exist today.

17. That so far as we know there is nothing in the origin of cancer that calls for a feeling of shame or the necessity of concealment.

18. That it will be promotive of good results if members of the public who are anxious about their health and those who wish to preserve it will, on the one hand, avoid assuming themselves to be sufferers from one or another dreadful disease, but, on the other hand, will submit themselves periodically to the family physician for a general overhauling.

19. That at all times under all conditions there is much to be hoped for and nothing to be feared from living a normal and moderate life.

20. That the finding of any abnormal condition about the body should be taken as an indication for competent professional and not personal attention.

21. That watchwords for the public until "the day dawns" and the cancer problem is solved, are: Alertness without apprehension, hope without neglect, early and efficient examination where there is doubt, early and efficient treatment when the doubt has been determined.

